# A Heterogeneous Wireless Identification Network for the Localization of Animals Based on Stochastic Movements

**DOI:** 10.3390/s90503942

**Published:** 2009-05-25

**Authors:** Álvaro Gutiérrez, Carlos González, Javier Jiménez-Leube, Santiago Zazo, Nelson Dopico, Ivana Raos

**Affiliations:** ETSI Telecomunicación, Universidad Politécnica de Madrid, Avd. Complutense 30, 28040 Madrid, Spain; E-mails: cgonzale@etsit.upm.es (C.G.); jleube@etsit.upm.es (J.J.L.); santiago@gaps.ssr.upm.es (S.Z.); nelson@gaps.ssr.upm.es (N.D.); ivana@gaps.ssr.upm.es (I.R.)

**Keywords:** stochastic transmission, energy harvesting, heterogeneous network, energy-aware network

## Abstract

The improvement in the transmission range in wireless applications without the use of batteries remains a significant challenge in identification applications. In this paper, we describe a heterogeneous wireless identification network mostly powered by kinetic energy, which allows the localization of animals in open environments. The system relies on radio communications and a global positioning system. It is made up of primary and secondary nodes. Secondary nodes are kinetic-powered and take advantage of animal movements to activate the node and transmit a specific identifier, reducing the number of batteries of the system. Primary nodes are battery-powered and gather secondary-node transmitted information to provide it, along with position and time data, to a final base station in charge of the animal monitoring. The system allows tracking based on contextual information obtained from statistical data.

## 1. Introduction

The effects of physical and biological factors on activity patterns and movements of animals are of major interest for biologists. A primary requisite for understanding certain aspects of animal behavior is knowing their location and mobility patterns. Tracking animals is important because they are often part of evolutionary and ecological experiments, they provide important ecosystem services and they are of conservation concern.

The use of mobile sensor networks promises a fruitful future for animal behavioral sciences, although some difficulties arise when trying to insert electronic devices in a natural environment. In most wireless sensor networks, power is a constraint from both the technological and the ecological point of view. On the one hand, the power supply is a severe constraint in achieving a long lifetime in a wireless autonomous embedded system. In a battery operated system, the system lifetime is directly related to the node battery lifetime. Moreover, in an animal tracking case, if thousands of animals must be monitored, the thousands of nodes must be battery operated. Therefore, high maintenance costs result and a tedious task of battery replacement will come up during a long term experiment. On the other hand, working in an outdoor large environment, the use of batteries might be harmful for the ecosystem. If we spread a large amount of battery-powered nodes throughout the environment, there is a great possibility that some nodes get lost, and therefore an unexpected pollution might arise.

A number of different strategies have been used to provide power to animal tracking systems avoiding battery restrictions (see Section 2). Although the use of battery-powered systems is probably not completely avoidable, researchers must try to develop systems where the use of batteries is reduced to a minimum.

Following these lines of research, we have pursued the implementation of a heterogeneous network for animal tracking which tries to minimize the use of battery-powered nodes. This minimization is achieved by taking advantage of kinetic energy. During the day, animals perform different actions which imply the generation of kinetic energy because of their movement. In the system proposed, this energy is converted into electrical energy by the network's nodes. The network is made up of secondary nodes and primary nodes. The former are kinetic-powered nodes which, by taking advantage of animal movement, are able to transmit minimal information, while the latter are battery-powered nodes which take care of secondary-node communication events, adding location and current time and transmitting it to a central monitoring system. We have not attempted to carry out a conclusive study of animals movement, but rather suggest a design method for mobile sensor networks which: *i)* saves battery costs, *ii)* is able to operate under sunlight restrictions and *iii)* takes advantage of animals' kinetic energy. Therefore, we pursue the implementation of a wireless sensor network designed to provide quantitative data on animal behavior under natural conditions.

The paper is organized as follows: Section 2 describes related work on harvesting systems and animal tracking. Section 3 sets out the network hardware, where the primary and secondary nodes are described. Experimental results are presented in Section 4. Finally, Section 5 concludes the paper and suggests future developments.

## 2. Related Work

In recent decades different systems have been designed for animal tracking. Some of them make use of satellites which locate the animal's position [1]. These systems allow the determination of the position of animals which have been equipped with a satellite emitting system. They have been widely used in turtle [2], duck [3] or whale [4] tracking. However, its use is extremely expensive and requires all the satellite transmitters in the animals to be updated in the satellite database. Moreover, satellites are not able to take more than some tens of measurements per day.

Other implementations are based on GPS devices which allow a larger data rate update [5]. However, commercially available tracking systems lack the data storage capacity needed to collect animal location data frequently over long-term deployment periods. Some commercial systems have remote data-download capabilities, reducing the need to recapture tagged animals for data retrieval. These systems download data via satellite, mobile communications (GSM) or radio communications. However, satellite systems are excessively expensive and GSM coverage is extremely limited in some areas within the Globe. Therefore, only radio communications or manual download are feasible for most of standard systems. Nevertheless, these battery-powered systems need to be replaced once exhausted and suffer from the disadvantages already mentioned in Section 1.

Different researchers are working on energy harvesting systems which allow animal tracking systems to take advantage of renewable energy, avoid battery replacement, or reduce its use to a minimum. When energy harvesting is applied to mobile sensor networks, the energy from external power sources can be used to power the nodes and increase their lifetime. Energy harvesting can be divided into two categories: *i)* energy to use and *ii)* energy to store. In the former, the system directly powers the sensor node and it continues operating until the harvester stops suppling energy. In the latter, a storage system (typically a battery or super-capacitor) is present and the energy produced by the system is stored in the battery for its later use. Systems which generate energy for its direct use are the ones that must be actively pursued for an ecological environmental operation. However, most of the systems found in the literature make use of storage systems.

Most of these strategies make use of solar energy as the environmental energy source [6, 7]. In [8], for example, a single-storage energy harvesting system based on solar energy is built on a Mica2 platform [9]. The energy obtained is used to recharge a AA-size Ni-MH battery of 1,800 mAh capacity. In [10], the authors presented a system based on a double storage method. The primary system is recharged using a solar cell. If there is an excess of energy in the primary system, it recharges the secondary system. When there is not enough energy in the primary system, the node switches to the secondary system for its supply until the primary system gets recharged again. In [11], a non-battery-powered node is presented. It uses a super-capacitor which is recharged by means of solar energy. The authors claim that the system is able to operate for 20 years at 50% duty cycle. In [12], the authors also make use of a super-capacitor which is recharged when some of the photodiodes are enabled because of the light detection. Some of these implementations have been used for animal tracking, as in the *zebranet* project [13] or the *Turtlenet* project [14]. However, solar energy is severely limited for latitudes where daily sunlight may be short in some seasons or the irradiance is not enough to power the system.

To deal with solar energy restrictions, some other techniques have been studied. In [15], the authors describe a system which takes advantage of human motion and obtains enough energy for transmitting information. The system is based on a piezo-electric film (*PolyVinylidene Fluoride*) which generates energy when the film is bent. However, the communication is based on an RFID tag transmitter which does not allow reading distances of more than just a few meters. Moreover, the system was used in a shoe and has only been tested on humans. In [16], the authors follow the same principle by taking advantage of finger motion. A piezo-electric system based on a push button is presented in [17]. The system is able to transmit 12 bit information within a range of 15 meters. Although piezo-electric systems presented in the literature try to avoid the use of batteries, the typical ranges achieved make these systems unsuitable for animal tracking in open environments. Some other strategies make use of wind energy [18] or radio frequency energy such as RFID system [19]. However, while the former are not useful for animal tracking systems because animals avoid wind flows, the latter suffer from poor emission ranges which make the system unsuitable.

## 3. Hardware Description

### 3.1. The Network

**Figure 1.**
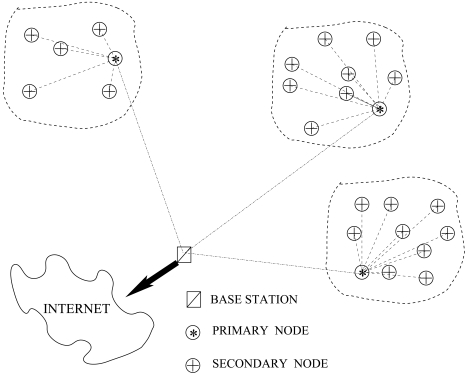
Network description.

The system goal is to create an identification network which is able to localize mammals in open environments. The network architecture proposed is made up of three different elements: primary nodes, secondary nodes and base stations (see Figure 1). Primary and secondary nodes are mounted on animals and therefore are mobile nodes, while base stations are static nodes. Secondary nodes are the simplest elements in the network. They take kinetic energy from the animal movements which produce just enough power to create and transmit a unique identification (ID) to the environment without confirmation of its reception. If a primary node is within the range of transmission, it receives and stores the transmitted ID. Moreover, primary nodes are able to obtain their global position thanks to a Global Positioning System (GPS) which can be switched on and off depending on the final application needs. Therefore, a primary node, which receives a transmission from a secondary node, approximates the secondary node position through its own location. Moreover, while the primary node is moving in the environment, it creates a table with the different secondary nodes IDs received, their approximated position and the time when the transmission was produced. When a primary node enters within the base station communication range, it downloads all the information acquired from the secondary nodes along with its own trajectory information. A base station is a static battery-powered node which has access to the Internet and is able to offer the data to a final monitoring system. Therefore, once the base station receives the data dumped by the primary node, it will send it to the monitoring system. This monitoring system will receive information from different base stations, hence it will merge all the information and supply it to the final user.

Following these principles, the final system has information about position estimates of primary and secondary nodes. Therefore, it will be able to reconstruct the movements carried out by the different animals. Notice, that this reconstruction is an approximation of the real trajectories followed by the individuals, because of the stochastic transmission of secondary nodes, the probabilistic reception of the primary nodes and the discretization of the GPS readings depending on the final application. As observed, the system provides a network structure to monitor animals in open environments. However, for its correct working in a specific and real application, some parameters such as the time the primary nodes are listening to the secondary nodes, the times a GPS connects to obtain its position, the ratio between primary and secondary nodes, or the density of base stations among others, must be defined for each specific application. Because secondary nodes are non-battery-powered nodes, simpler and cheaper than primary nodes, the designers' goal should be to try to reduce the number of primary nodes and base stations in favor of secondary nodes.

For the correct understanding of the network, the forthcoming subsections detail the different hardware nodes implemented.

### 3.2. Secondary Node

As previously explained, secondary nodes act as active IDs beacons. Each secondary node consists of two different parts: a kinetic harvester module and a transmitter module. The former is in charge of generating power produced by the animal's movement while the latter is in charge of transmitting a unique identified ID.

#### Kinetic energy harvesting module

**Figure 2.**
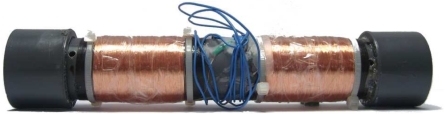
The magnetic kinetic generator module.

The kinetic module is divided into a magnetic-coil generator and a regulator circuit. The generator is designed with a cylindric tube with two coils located at each end of the tube and a “Neodymium-Boron-Iron” magnet inside the tube (see Figure 2).

**Figure 3.**
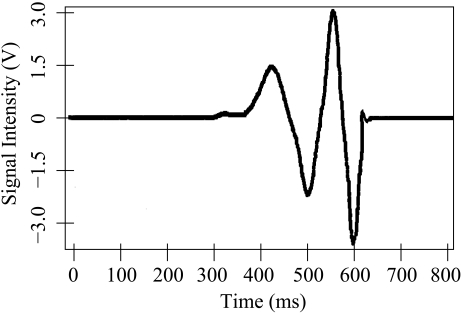
Waveform at the output of the magnetic kinetic generator module, after a full swing.

When the generator is tilted, the magnet moves through the coils and it generates a voltage which follows Lenz law As the magnet goes through one of the coils, a voltage cycle occurs. Figure 3 shows the voltage waveform at the coil output in a swing of the magnet. Notice that the magnet starts at one end of the tube, and passes through the first coil (first positive cycle), moves out of the first coil (first negative cycle), passes through the second coil (second positive cycle) and finally comes out of the second coil (second negative cycle).

**Figure 4.**
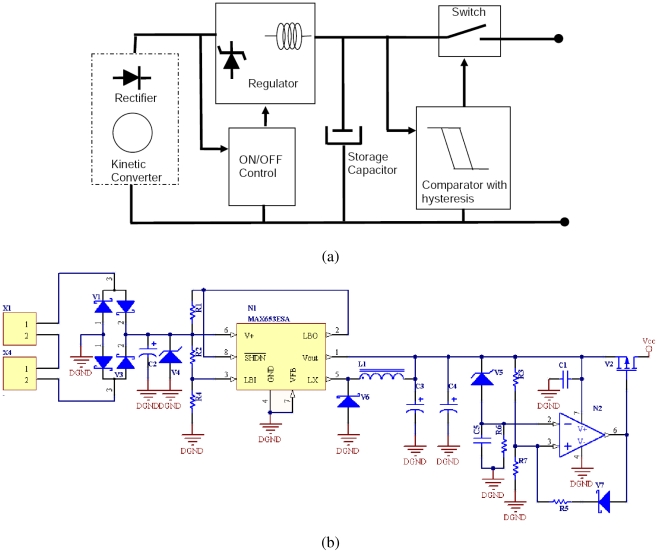
(a) Block and (b) Schematic diagram of the kinetic harvester module.

Once the power is available at the input of the regulator circuit, it has to be rectified and regulated before powering the transmitter. A block and schematic diagram are detailed in Figure 4.

The energy supplied by the generator is first rectified by a diode bridge. Four Schottky diodes are used to create the rectifier bridge; a voltage drop of about 0.25 V per diode is observed. A 100 *μ*F capacitor and a 10 V limiting zener diode are located at the output of the bridge to protect the next stage input. The regulator circuit adapts the input voltage to a steady stage voltage. A step-down dc-dc converter is used to stabilize the output to 3 V. The regulator charges two 100 *μ*F capacitors in the short bursts that correspond to the existence of energy at the input (a magnet swing) to the desired dc voltage (3V).

After the regulator, a control circuit with hysteresis is designed to ensure enough energy for a frame transmission. Two different hysteresis thresholds are obtained thanks to a Schottky diode at the output to guarantee stability. Therefore, a nano-power comparator is used to ensure no energy is supplied to the transmission module until a minimum voltage is accumulated in the storage capacitor (high level hysteresis threshold). Once the comparator enables the output, it keeps energy delivery on until the storage capacitor voltage drops below a minimum threshold (low level hysteresis threshold). The output control is managed with a pMOS transistor which acts as a switch. When the comparator allows the energy delivery, it closes the switch, and when a given threshold is reached, the comparator opens it again. Duration values of power pulses are typically above 20 ms, which allows more than one frame transmission as explained in the following section.

#### Transmission module

The secondary node's communication is designed to operate in the 433 MHz ISM open band. The band was selected because: *i)* it is an open band that can be used without licence, provided that some regulations are met, *ii)* commercial low cost IC chips are available for the ISM band, *iii)* propagation path loss is lower as the frequency decreases and *iv)* the size of the antennas is reasonably small for a miniaturized node.

**Figure 5.**
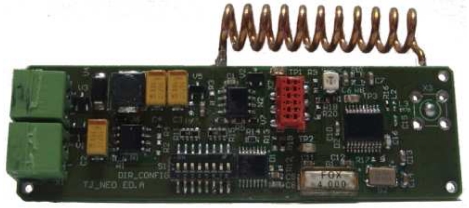
Secondary node.

The transmission module consists of a radio frequency transmitter/controller with an 8 bit ID selection switch (see Figure 5). The modulation selected is binary FSK with a frequency deviation of about 25 kHz coded in Manchester. When the power burst is available, the MCU initializes, loads the ID, turns on the RF emitter, waits for a stable signal and transmit the frame. For the prototype testing, the frame rate was selected to comply with a 240 *μ*s bit period. The selected value gives a raw symbol rate of about 8 kbps and a data rate of about 4 kbps, because of the Manchester encoding. The frame is made up of 8 ON/OFF transmission periods to allow synchronization, a 2 bit start word, 1 byte of data and 1 byte of CRC, where the data byte contains the node ID. Therefore the transmission of a frame lasts for 6.24 ms. Using the ID described, a maximum of 256 nodes are tagged. However in a final production design, the ID node should be flash-programed. Therefore, the ID could be increased up to the duration value power pulses (typically above 20 ms).

Finally an adapted antenna has been added. We have chosen a pig tail antenna (see Figure 5) which allows the design of the board to be thinner than by using a standard monopole. Because future experiment expectations will involve mammals, we wanted the secondary node to be as least uncomfortable as possible for the animals, without reducing range transmission capabilities. As shown in Figure 5, both the harvesting and transmission modules are located in the same board.

### 3.3. Primary Node

**Figure 6.**
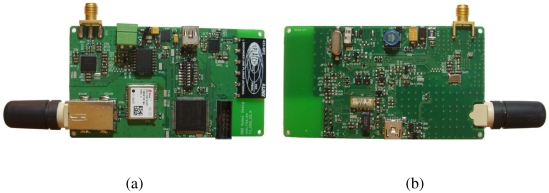
(a) Top and (b) bottom view of the primary node.

The primary node is an autonomous battery powered system controlled by its own processor (see Figure 6). It manages the secondary node's communication reception and the exchange of information with the base station. The node is equipped with a GPS, a 433 MHz receiver and a 166 MHz transceiver.

The primary node is governed by an ARM-7 microcontroller selected because: *i)* its low cost and consumption, *ii)* the internal peripherals can be independently controlled and therefore the consumption can be adjusted to the needs of the peripherals, *iii)* it allows the implementation of a real time operating system, *iv)* it is a well studied architecture and *v)* it has a GCC compiler.

Moreover, the board has been designed in such a way that all external peripherals (i.e. GPS and radios) are power-controlled by the microcontroller. When no peripheral is active and the microcontroller is in sleep mode, a minimum consumption of 20 *μ*A at 3.3 V is achieved. For each peripheral, the typical consumption when active is shown in Table 1. The board includes a 64 Mbit flash memory controlled through an SPI bus. This memory is in charge of storing the information provided by the secondary nodes for its transmission to the base station. In the board's design we have actively pursued *i)* low consumption and *ii)* miniaturization. Therefore, each of the internal modules explained below tries to follow the aforementioned characteristics. Moreover, because of the miniaturized components, the board has a size of 9×5 cm^2^ which fits the specifications of being carried by the target mammals.

A *u-blox* GPS module is integrated into the board. This GPS module allows a connection time of less than one second which reduces the position acquisition and therefore saves power. It has a -160 dBm acquisition and tracking sensitivity and it is highly immune to jamming. Its low consumption characteristics and miniature size (16.0 × 12.2 mm^2^) make the module suitable for our application. Moreover, the module allows several connectivity options (i.e. UART, SPI, USB and I2C). In our design, the controller communicates with the GPS module through a UART while the USB connector has also been introduced for possible extended developments.

An FSK superhetorodyne dual-channel receiver has been introduced into the design for the communication with the secondary node. It is tuned to receive at a frequency of 433 MHz and includes a low noise amplifier, an image-reject mixer, a PLL, a local oscillator, a limiting amplifier, a low-noise FM demodulator and a 3V voltage regulator. The module can be switched on and off by the microcontroller to save power. Preliminary results show that the primary-secondary node communication achieves ranges of up to 100 m (see Section 4). A patch antenna as been added to the communication system which allows high communication ranges.

The long range communication system is implemented with an ISM Band FSK transceiver. It is designed for operation in the low UHF and VHF bands and a 166 MHz band has been chosen. In the primary-base station communication we emphasize maximum range, simple interface and high data rates. The transceiver allows a frame rate of up to 200 kbps and has a -119 dBm sensibility. Moreover, the transceiver allows a programmable output power from -16 dBm to +13 dBm in 0.3 dBm steps. The antenna should be located on the top of the supporting animal, therefore an SMA connector has been added for the 166 MHz antenna. This connector allows the antenna to be wired to an upper position, for example in a specific collar.

### 3.4. Base Station

For design simplicity, the base station and primary nodes are designed on the same printed circuit board. The only difference between the base station node and the primary node is a USB interface which allows communication with an embedded computer. Therefore, when the information from the primary node is received by the base station, it communicates with the computer to download all the data. In the primary node, the USB interface is not connected.

Because the base station is assumed to be in an area where electric power is present, it always keeps its radio transceiver on, waiting for any primary node to transmit the information. Moreover, it also keeps its 433 MHz radio interface enabled, so if any secondary node is within the communicating range it stores its ID together with the current time and its static position, as if it were a primary node. Because the base station is intended to be a static element of the network, it always has a predetermined global position. Therefore, the GPS module is not present in the board, saving costs and power.

## 4. Experimental Evaluation

Different experiments have been run to characterize and validate the localization and communication systems. The first experiment is developed in an obstacle-free environment with a secondary and a primary node to test the stochastic 433 MHz link. The second experiment is developed with a primary node and a base station node in an obstacle-free environment to analyze the 166 MHz link over the implementation of a specific protocol. Finally, an experiment involving the GPS has been carried out to estimate the time required for the GPS to obtain the current time and position.

### 4.1. Primary-Secondary nodes radio link experiment

**Figure 7.**
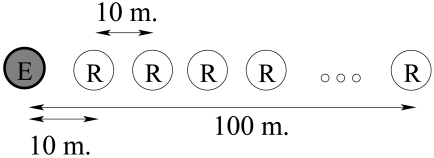
Physical arrangement of nodes for the Primary-Secondary radio link modelling experiment. Figure not to scale.

One secondary node and one primary node are placed in an obstacle-free environment from 0m to 120 m in 10 m intervals as shown in Figure 7. At each distance the secondary node generator is swung 30 times and therefore 30 frames are transmitted. Each time the primary node receives a frame it is stored in the EEPROM memory for its download in a PC computer. We have repeated this test for 12 different positions and 6 different nodes.

**Figure 8.**
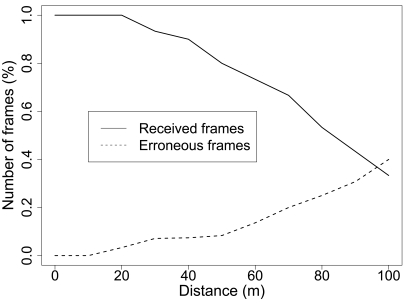
Average number of frames received and erroneous messages in a Primary-Secondary radio link experiment for all the distances.

Figure 8 shows the error rate average of the communication system. We observe that for short distances (less than 20 meters) all the frames transmitted arrive correctly at the primary node. When the emitter moves away from the receiver, some frames are not received and get lost. Only 30% of the frames arrive at the receiver at 100 m and no frame is received at larger distances. Moreover, we observe after 30 m some of the frames received are not correctly decoded, with a maximum error of 40% when the nodes are at their maximum transmission range.

### 4.2. Primary-Base station nodes radio link experiment

**Figure 9.**
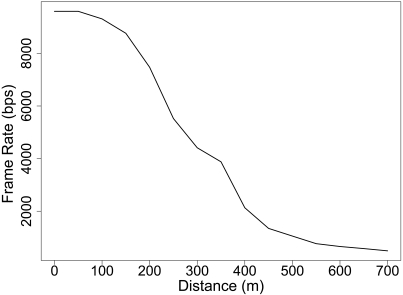
Average frame rate achieve in the Primary-Base station radio link experiment for different transmission distances.

A similar test is carried out with the Primary-Base Station radio link. One primary node and one base station node are placed in an obstacle-free environment from 0 m to 700 m in 50 m intervals. At each distance, the primary node transmits 30 messages which are received by the base station. The Primary-Base station radio link implements a communication protocol which is in charge of collisions. Therefore, all the frames transmitted by the primary node are received by the base station if it is within the communication range. However, because of the distance, some messages get lost and the primary node must retransmit them until it receives an acknowledgement from the base station. The test messages to be transmitted are made up of 6,000 bits. In a perfect link transmission a frame rate of 9,000 bps is achieved, because of the header and CRC load. However, we observe in Figure 9 this frame rate gets reduced because of the retransmission handled by the protocol. A maximum transmission range of 720 m has been achieved. We have repeated this test for 15 different positions and four different nodes.

### 4.3. Localization and timing experiment

We have carried out an analysis of the GPS performance. The GPS selected is able to provide the current GMT time, latitude and longitude. We tested the whole GPS system for a coldstart configuration. A coldstart is when the GPS has been powered off for a long period and then it is switched on. At this moment, the GPS needs to find the satellite's position to locate itself. The first data the GPS obtains is the satellite connection and the current time. Typically, 5 to 20 seconds are needed for the timing acquisition. After this first period, the GPS obtains the satellite's positions and once different satellites have been located, the GPS is able to obtain its own location. A period of 2 minutes on average is needed for localization after a coldstart. Once located, the GPS updates its position every second.

### 4.4. A live animal test

Finally, we made a simple proof-of-concept study of the network in live animals. We have already discussed that we were not attempting to carry out an animal location study, but to provide the tools for a future use. However, before providing these tools to researchers in charge of studying animal behaviors, we run some simple tests in animals to validate our system. The main idea for the animal test was to check *i*) if animal movements were able to swing the generator to produce enough energy for the ID transmission and *ii*) if the node size and dimensions are feasible at least for some mammals.

**Figure 10.**
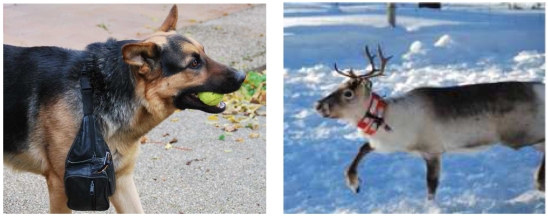
A secondary node (a) in a hip bag around a dog neck in an Spanish courtyard and (b) mounted on a reflective collar around a reindeer neck in Northern Sweden.

We tested our system both in a dog and a reindeer. The best place for the secondary node location, turned out to be the animal neck (see Figure 10). When the animals move their neck (for example, because of a movement produced when looking for food on the ground), the generator is swung. The neck was selected because, to best take advantage of the energy, the generator needs to be swung from end to end. We also tested to adjust the secondary node to the legs and body of the animal. In this case, faster and more frequent movements were achieved. However, the magnet does not move along the whole generator. Typically, it did not cross the two coils, hence it did not produce enough energy for a frame transmission.

During the trial, two generators were used to power the secondary nodes, although it was noticed that the transmission was also achievable with just one generator. Communication ranges of over 100 m have been also observed in an open area when the kinetic converter was activated by the animal movement. Average data transmissions rates of one frame every three minutes were estimated from the first results depending on the animal's activity. The modules were operationally tested at temperatures as low as -12 °C in Northern Sweden, although they were also tested in a cold experimental chamber at temperatures of -25 °C. Moreover, all of the node's components are rated at -40C, for its correct operation in different test environments.

## 5. Conclusions

In this paper we have described a heterogeneous mobile sensor network for animal tracking made up of battery-powered and kinetic-powered nodes. The system tries to minimize the use of battery-powered nodes to a minimum which satisfies future real animal experiments and provides the means for animal localization. The system is based on the assumption that animals carrying kinetic-powered (secondary) nodes are tracked by their neighbors and their positions are approximated by the ones carrying battery-powered (primary) nodes. Therefore, primary nodes keep track of the information sent by secondary nodes and can download it to a base station when they enter its coverage range.

Secondary nodes have been designed with a 433 MHz transmitter powered by a kinetic converter system made with coils and magnets. Primary nodes implement a 433 MHz receiver to gather secondary node information, a GPS to obtain its position periodically and a 166 MHz transceiver to communicate with the base station. Moreover, the primary node and base station communication interface has been designed over the same printed circuit board for cost saving. The system has proved to achieve a communication range of 100 m on the secondary-primary node communication and 700 m for the primary-base station link.

We have tested the system in an animal environment (i.e. dogs and reindeers) showing good performances. In the future, we intend to introduce the system in a herd of reindeer in Northern Sweden to study the relationship between primary and secondary nodes needed to keep track of the herd and study its behavior. However, depending on the animal behavior and the requisites of the experiment, different parameters, such as acquiring data frequency of the GPS, the 166 MHz radio working cycle, or the time the primary node must listen to a secondary node, should be studied before testing the system in a real scenario.

## Figures and Tables

**Table 1.** Consumption characteristics of the primary node.

Working mode	Peripheral	Consumption (mA @ 3.3V)
Sleep	NONE	20 *μ*A
Standard	NONE	20 mA
Standard	GPS	150 mA
Standard	433 MHz radio	22 mA
Standard	166 MHz radio	146 mA
